# Management of the third stage of labour by Basotho traditional birth attendants

**DOI:** 10.4102/hsag.v29i0.2372

**Published:** 2024-03-20

**Authors:** Keneuoe N. Fobo, Gaotswake P. Kovane, Catharina S. Minnie

**Affiliations:** 1 School of Nursing, Faculty of Health Sciences, North-West University, Potchefstroom, South Africa; 2 School of Nursing, Faculty of Health Sciences, North-West University, Mafikeng, South Africa; 3 School of Nursing, Faculty of Health Sciences, University of the Western Cape, Cape Town, South Africa

**Keywords:** placenta, home delivery, traditional birth attendant, third stage of labour, postpartum haemorrhage

## Abstract

**Background:**

Historically and to date, women still give birth at home with the support of elderly, experienced women who live within their communities. In Lesotho, traditional birth attendants (TBAs) are sometimes the only option for pregnant women living far from facilities. Women are vulnerable during the third stage of labour; therefore, correct management is crucial to limit undesirable outcomes. Postpartum haemorrhage and postpartum sepsis remain the leading direct causes of maternal mortality.

**Aim:**

This study aimed to explore and describe how Basotho TBAs manage the third stage of labour.

**Setting:**

The study was conducted in Lesotho, at Bolahla and Sejakhosi. These villages have the highest number of women giving birth at home.

**Methods:**

An explorative and descriptive design with a qualitative approach was used. Semistructured interview guide was utilised to conduct individual in-depth interviews about how the TBAs manage the third stage of labour and their support needs concerning this phase. The TBAs were purposively sampled. The data were analysed thematically.

**Results:**

Four themes emerged: challenges TBA experience in the management of the third stage of labour, management of the placenta by Basotho traditional birth attendants, support during the management of the placenta by Basotho traditional birth attendants, and management during emergencies.

**Conclusion:**

This study demonstrated that if TBAs are supported, they can contribute to the health of the mother and baby.

**Contribution:**

This study’s findings can be valuable to healthcare professionals to understand better how TBAs in Lesotho manage the third stage of labour and the support they need.

## Introduction

In rural communities across the globe, before the arrival of modern obstetric services, traditional birth attendants (TBAs) have been providing maternity services to women during pregnancy, childbirth and after birth. They were the principal caregivers for women during delivery and after delivery of the baby (Aziato & Omenyo 2018:19; Garg & Bansal 2020:143; Miskinzod 2021:480), especially in Africa. The World Health Organization (WHO 2015) defined a TBA as ‘a person who assists the mother during childbirth and initially acquired her skills by delivering babies herself or through apprenticeship to other traditional birth attendants’. They are not recognised as medical practitioners or ‘skilled birth attendants’ because they do not have any formal training (Garces et al. 2019:247). However, TBAs continue to play a significant role in maternal health care in rural and deprived communities such as Lesotho (Adatara et al. 2018:354; Hobday et al. 2018:174).

Skilled birth attendants are health workers who have acquired their skills through professional training to render maternal and newborn care, and are regulated by national and international standards (WHO 2018). Although care provided by a skilled birth attendant is identified as critical for saving the lives of pregnant women and infants, in rural and deprived communities in developing countries, women continue to deliver at home with the assistance of TBAs (Cheptum et al. 2017:56; Ibrhim et al. 2018:51; Wilunda et al. 2017:e0185726). Home delivery without a skilled attendant is a reality for women in rural and remote areas of low socio-income countries, despite having policies to achieve the highest possible coverage of facility births with skilled birth attendants (Aziato & Omenyo 2018:18–64; Ngunyulu, Mulaudzi & Peu 2020:2–3). Most governments are phasing out TBAs in anticipation of increasing skilled birth attendants who will be able to manage complications, hence decreasing the risk of maternal and perinatal mortalities (Garces et al. 2019; Garg & Bansal 2020:143; Ntoimo et al. 2022). Despite the government’s efforts, many women in low-resource settings continue to lack access to adequate health services because of diverse reasons. Therefore, they prefer to give birth at home rather than in a health facility (MacDonald 2017).

In low- and middle-income countries where maternal mortality ratio (MMR) continues to be inappropriately high, governments are again starting to recognise the importance of training TBAs to help improve outcomes during pregnancy and childbirth (Hernandez, Oliveira & Shirazian 2017:111). Low-income countries such as Lesotho, which have economic and logistical barriers to providing enough skilled birth attendants, and where some women live in remote areas with inadequate transport to health facilities, could benefit from a training programme (Hodnett 2012:e365; Wilson et al. 2011:343).

Competent trained staff is a necessity for the management of the third stage of labour as it is very risky for the birthing woman. This is a time when the placenta and membranes are delivered. In low-resource settings, even a blood loss of 500 mL after delivery can be harmful. Therefore, active management of the third stage of labour (AMTSL) is frequently advised for all births in order to decrease blood loss after delivery (WHO 2012:1). Active management of the third stage of labour consists of three parts: a uterotonic, preferably oxytocin, should be administered right after the baby is born, followed by controlled cord traction to deliver the placenta and massage of the uterine fundus. Active management of the third stage of labour is not always practical in contexts with limited resources. The majority of uterotonics, like oxytocin, necessitate parenteral delivery, sterile equipment, competent staff and cold storage. Oxytocin prophylaxis is, therefore, mostly limited to facility-based deliveries and to those attended by a professional provider, where the cold chain can be maintained, leaving the majority of deliveries in community settings with no uterotonics coverage (Mobeen et al. 2010:354).

Bell et al. (2014:118) investigated the effects of various interventions to prevent postpartum haemorrhage at home births in Bangladesh. The attendant can also manage the third stage of labour expectantly and/or physiologically without medication. When the placenta is delayed being delivered, but the mother is not losing much blood, the attendant should not do anything, and the cord must not be pulled as this can cause a dangerous haemorrhage.

Postpartum obstetric haemorrhage is one of the major causes of maternal mortality among Basotho women (Ministry of Health [Lesotho] and ICF International 2016, 2014:123). A significant number (23%) of women in Lesotho still give birth at home even though there is an increase in facility delivery from 59% in 2009 to 77% in 2014 (Ministry of Health [Lesotho] and ICF International 2016, 2014:126).

In the absence of appropriate local evidence, it is not possible to know how TBAs in Lesotho manage the third stage of labour and how they could be supported to manage the third stage of labour. Therefore, it was essential to explore and describe Basotho TBAs’ ways of managing the third stage of labour and how they can be supported to manage the third stage of labour safely.

## Research methods and design

An explorative and descriptive design with a qualitative approach was employed, focusing on individuals’ stories (Creswell & Poth 2018:143). This design was adopted to guide the researcher to explore and describe how Basotho TBAs manage the third stage of labour.

### Study setting

The southern part of Quthing District has 10 villages belonging to the Nkuebe Community Council (G05) under the chieftainship of Mrs. Tlalane Seleke. For this study, two villages were chosen: Bolahla and Sejakhosi, as they are the most remote villages which have the highest number of women giving birth at home. These villages are served by St Gabriel Catholic Church Health Centre, which according to the chief, is the nearest health facility which is 10 km away from the villages. People travel on foot for 6–8 h to reach the health facility. The Nkuebe Community Council has about 10 487 people, which is 8.8% of the total district population (Ministry of Local Government and Chieftainship [MoLGC] 2008).

### Sampling technique

Purposive sampling was used to recruit the sample of TBAs who participated in the study. Traditional birth attendants were included in the study if they learned how to assist women during childbirth from another TBA, had assisted at least three women during childbirth, lived in the selected village, and were able to speak Sesotho or English. Potential participants who did not meet the set criteria were excluded. The sample size for this study was guided by data saturation when no new information was emerging from the interviews. In all, 9 TBAs participated in the study.

### Data collection

Semi-structured interviews were conducted to collect data. Interviews lasted 30–60 min, and most took 45 min. The duration of data collection was from 07 July 2022 to 29 July 2022. The researcher started the interview session with a small talk to ease participants before commencing data collection. A semi-structured interview guide was used, and each participant was encouraged to talk freely about how she manages the third stage of labour in her preferred language. During the interviews, the interviewer used several communication methods to maintain rapport. Such methods included making eye contact, using an open posture, and displaying a non-judgemental attitude and respect. The interviewer practised active listening, using minimum verbal and non-verbal responses. Open-ended questions, prompts and probes were used to guide the interview to ensure that all areas of the research issue were covered and to encourage the respondents to elaborate on their responses (Polit & Beck 2017:624).

### Data analysis

The narrative data from the semi-structured interviews were analysed using a thematic analysis approach as indicated by Kumar (2011:152). The interviews were transcribed verbatim by repeatedly listening to the audio tapes to capture the content accurately. The transcriptions were translated from the local language (Sesotho) to English and back to Sesotho to confirm if the meaning was consistent with the response received from the participant. The researcher repeatedly read the transcripts and categorised them. Categories with similar meanings were grouped, and themes were then formulated to represent the grouped categories. An independent coder, experienced in qualitative research, independently coded a copied set of raw data. Meetings were held between the researcher and the independent coder to compare and verify themes and categories that had emerged between the two analyses.

### Ethical considerations

Ethical clearance was obtained from the Human Research Ethics Committee (HREC) of the North-West University (Ethics Number: NWU-00250-21-A1). The researcher obtained permission to conduct the study from the Lesotho Ministry of Health. Verbal consent to conduct the study was also obtained from the chief of the two villages where data were collected. Furthermore, informed consent was obtained from each participant without coercion, including permission to audio record. The research participants’ confidentiality was maintained by ensuring that data were not linked to any participant’s name through use of anonymous codes. All soft copy data were safely encrypted with a password, and hard copies were locked in a cupboard.

### Measures to ensure trustworthiness

To ensure the credibility of the study findings, a detailed description of the study setting, components of the population, and all steps taken was provided. The researcher and the trained field worker spent time with participants for a better understanding of their practices of management of the third stage of labour. The interview guide, transcriptions and field notes are available for auditing to establish the conformability of the findings.

The researcher collected well-detailed and sufficient descriptive data that included comprehensive descriptions of the participants’ characteristics and settings to enable readers evaluate the applicability of the data to other contexts in similar situations (Creswell & Poth 2018:343). The quotes from the participants are also provided in the data analysis. Rich descriptions of the procedure and findings are provided to ensure transferability.

The dependability was ensured by examining study’s findings, interpretation and recommendations to ensure they are all supported by the data collected from study participants. The researcher and the field worker kept a record of what they did during data collection and analysis to ensure that the protocol was strictly followed. To ensure and confirm the correctness of the data collected, the researcher did a test-and-retest.

## Results

The participants in the study were mainly older females who have delivered women from home and had no formal training to conduct deliveries; they mainly learnt the skills from observing other TBAs. Extracts from the TBA interviews have been used to support descriptions of the themes. Grammatical modifications were made for clarity.

The following questions were asked:

- How do you deliver the after birth?
- How can you be supported in the way you support a woman directly after the baby is born?

Table 1 provides a summary of the themes and sub-themes.

**TABLE 1:** Themes and sub-themes.

Themes		Sub-themes
1.	Challenges TBA experience in the management of the third stage of labour	1.1 Women hiding their pregnancy 1.2 Distance from health facilities 1.3 No support from the department of health
2.	Management of the placenta by Basotho TBAs	2.1 Strategies to assist with the delivery of the placenta 2.2 No standard timing for delayed placenta expulsion 2.3 Measures taken when the placenta is retained and to ensure it is completely delivered 2.4 Assessment and management of blood loss after the delivery of the placenta.
3.	Support during the management of the placenta by Basotho TBAs	3.1 Support available during the management of the placenta 3.2 Support needed to ensure safe management of the placenta
4.	Management during emergencies	4.1 Measures taken by the Basotho TBAs during an emergency

TBA, traditional birth attendant.

### Theme 1: Challenges regarding the management of the third stage

The participants reported that there are some challenges that arise during the third stage of labour and few are related to some clients who hide their pregnancy. The participants raised a concern that this could lead to the death of a woman as complications that could have been identified early antenatally only get to be noticed at the time of delivery.

#### Sub-theme 1.1: Women hiding their pregnancy

It was reported that one of the challenges that TBAs faced was women’s non-disclosure of pregnancies on time. They verbalised that some women hid their pregnancies, resulting in them not attending antenatal care services where some high risk concerns could have been identified at the time. Most pregnant women sought assistance only when they were about to deliver the baby:

‘Sometimes there are women who hide their pregnancies, and they only come to us when they are sick, and they never indicate that the placenta is not all born, and because some women die, we only suspect that the placenta did not come out when we see the abdomen of the diseased extended.’ (P1, Female, 63 years)

#### Sub-theme 1.2: Distance from health facility

Another challenge was distance of health facilities from where pregnant women resided. This resulted in some women, especially those who experienced retained placenta, dying on the way to the health facility. It was reported that women often travelled long distances of more than 8 h on foot or horseback before reaching a health facility:

‘Situations where really the placenta refuses to come out even after trying all sorts of things, in the end, such a woman will be transferred to the health facility though they are very far, and sometimes women lose their lives with the placenta still inside.’ (P1, Female, 63 years)

‘We send the woman to the hospital if the placenta refused to come out, we wrap a stone with the umbilical cord and remove the reed so that nothing happens to this woman on the way to the hospital.’ (P8, Female, 68 years)

‘There should be a clinic nearby. We are living in the mountains far from the clinics, and when we encounter problems, we have nowhere to refer our women immediately.’ (P1, Female, 63 years)

‘We stay very far from the clinics.’ (P5, Female, 70 years)

### Theme 2: Management of the placenta by Basotho traditional birth attendants

Basotho TBAs have different ways they manage the third stage of labour. This theme had four sub-themes.

#### Sub-theme 2.1: Strategies to assist with the delivery of the placenta

Indigenous strategies were implemented for the successful delivery of placenta. The birthing woman is requested to blow into an empty bottle strongly:

‘A bottle is used for the woman to blow air into it strongly.’ (P1, Female, 63 years)

‘A bottle to blow air into it, like puuuuu, and she has to do it very strongly, and this time the placenta is expected to come out.’ (P7, Female, 44 years)

Traditional birth attendants also gave the birthing woman *Mooa* (black dust from the roof), which is believed to facilitate the expulsion of the placenta. The birth attendant mixes *Mooa* with warm water, strain it through a cloth and gives it to the woman to drink:

‘We give her Mooa to drink and it will eventually come out.’ (P2, Female, 62 years)

‘I normally mix this Mooa with warm water and sieve it through a cloth and give it to the woman to drink; it is this medicine that pushes the placenta out of the womb.’ (P3, Female, 59 years)

‘You take that Mooa fast and mix it with warm water and use a tablecloth to strain the unwanted materials and give it to the woman to drink it immediately.’ (P7, Female, 44 years)

The participants also encourage the woman to induce vomiting, which they believe tightens abdominal muscles. This is evident in the following quotes:

‘We take a string or anything and put it in the woman’s mouth to make her vomit; as she vomits, the placenta will also come out.’ (P2, Female, 62 years)

‘We take a string and put it inside the woman’s mouth’. I normally break one egg into a cup and mix it well and give it to the woman to drink to make the woman feel like she wants to vomit.’ (P3, Female, 59 years)

‘We normally insert something like a shoelace or string at the back of the mouth so that the woman could feel irritated and feel like vomiting.’ (P4, Female, 62 years)

‘To insert her two fingers into her mouth and induce vomiting.’ (P5, Female, 70 years)

‘I put my fingers firmly on the woman’s abdomen and ask the woman to breathe in and out like hmmm, hmmm. I use an egg and stir it well and give it to her to drink, the purpose of the egg is because it is slippery, it is expected to assist the placenta to just slip and come out.’ (P8, Female, 68 years)

Compressing the abdomen is another strategy used to assist in the delivery of the placenta:

‘The woman is pressed on the abdomen, then when that is done, the placenta will simply move down and come out.’ (P1, Female, 63 years)

‘We tie her just above the umbilicus and start to make mild compresses on the abdomen.’ (P2, Female, 62 years)

‘By compressing the woman on the abdomen with two hands, to assist the placenta to be born.’ (P3, Female, 59 years)

‘I will press the woman’s abdomen while she continues to push.’ (P6, Female, 74 years)

‘You press or compress the woman on the abdomen with your hands or fingers and try to press harder.’ (P7, Female, 44 years)

‘We rub the abdomen.’ (P9, Female, 66 years)

When the placenta delays, the TBAs also use grass called *Loli* to roll the umbilical cord around it, and start calling the placenta by blowing a whistle with the mouth (pipipi, pipipi, pipipi) – at the same time the umbilical cord is pulled down slowly:

‘We use a grass called Loli, and start touching the umbilical cord, and you do so you start calling the placenta by blowing a whistle with the mouth [*pipipi, pipipi, pipipi*] as you do so the placenta will actually move down.’ (P3, Female, 59 years)

#### Sub-theme 2.2: No standard timing for delayed placenta expulsion

Participants indicated different times allowed to birth the placenta if it is not expelled immediately after the baby is born:

‘I think about an hour, not more, failing which we will decide whether to transfer her or not.’ (P1, Female, 63 years)

‘I think 10 minutes is enough for one to see that the placenta is born.’ (P3, Female, 59 years)

‘We take something like 10 minutes to see if it is not coming, about 30 or more minutes, that is when I can be worried.’ (P4, Female, 62 years)

‘If it takes about 15 to 30 minutes, that is the time when I can really get worried.’ (P7, Female, 44 years)

‘We expect the placenta to follow the birth of the baby immediately after the baby is born, or about 10 minutes or more.’ (P9, Female, 66 years)

‘We make sure that by the time we finish cutting and separating the baby, the placenta should also be out.’ (P2, Female, 62 years)

#### Sub-theme 2.3: Measures taken when the placenta is retained and to ensure it is completely delivered

Participants indicated that, if the placenta did not come out after using all the strategies, they resorted to giving oral traditional medications. The common medications used are *Qobo* (wild rhubarb) and *Khomo ea Balisa (Bulbine narcissifolia):*

‘The woman is given a traditional medication called *Qobo* to drink, and that will assist the womb to expel all the clots that were left behind, once you see such clots, that means the placenta is all born.’ (P1, Female, 63 years)

‘We normally cook a medication called [*Khomo ea Balisa*], we cook it and give it to the woman to drink while it is warm, and this medication is the one that normally helps to remove those small pieces.’ (P2, Female, 62 years)

Some reported that they looked for signs such as shape and colour of the placenta and completeness of membranes to rule out retained products of conception. They also look at the shape of the placenta and that it does not appear ragged:

‘It is very easy to see it because on it there are some things that usually are hanging towards the end, and when you insert the finger into the vagina, nothing will come out.’ (P2, Female, 62 years)

‘When I see all those coverings of small meats are out.’ (P3, Female, 59 years)

‘We look at its shape, and normally it looks like a liver by shape and colour, so when I see that shape, I always make a conclusion that it is complete.’ (P4, Female, 62 years)

‘It is easy to see when it is complete, its round in shape and we look at all its corners, and if something is missing, we are able to see it.’ (P5, Female, 70 years)

‘We know it is complete and it does not appear ragged.’ (P6, Female, 74 years)

‘We actually put it in clean water and rinse it, and throw away that water, and do it again and again until that water is clean, then we check it to see if there is anything left behind.’ (P7, Female, 44 years)

#### Sub-theme 2.4: Assessment and management of blood loss after the delivery of the placenta

Participants indicated that positioning a woman during the process of birthing was very important to avoid tearing; thereby, preventing excessive blood loss.

‘The woman squats on sheep skin, and during squatting, she is also resting on what we call *Khare* [*a ring shape they make with a cloth*] which is placed between her heels and the anus so that she does not tear and bleed, if she has lost too much blood, she will complain of dizziness.’ (P2, Female, 62 years)

‘When the woman gives birth a sheep skin or an old blanket is placed underneath as she will be squatting, and normally we prepare a cloth and roll it to make it round and put it between the woman’s heels and the anus to prevent her from tearing during birthing and that alone will prevent her from bleeding.’ (P4, Female, 62 years)

Participants indicated that traditionally, a woman would be considered to have lost too much blood when the blood overflowed out of the blanket or the sheep skin she was lying on and when she bled continuously:

‘We use either an old blanket or sheep skin for the woman to lie on during birth, it is very easy to see when everything is too soaked that it is just too much.’ (P3, Female, 59 years)

‘You will realise the soaked linen with blood, and after changing that linen, if it becomes soaked, again and again.’ (P7, Female, 44 years)

‘We also look at how soaked is the blanket that this woman is sleeping on.’ (P8, Female, 68 years)

‘We see blood overflowing the area where the woman has been sleeping, and also if she bleeds continuously, that is when we realise that she has lost too much blood.’ (P9, Female, 66 years)

Some participants indicated that they were aware of signs that indicate that a women has lost too much blood, which include weakness, dizziness, blurred vision and shaking:

‘When they have lost too much blood, become weak and the weakness will be seen when you ask her to stand or move from the area of birthing that she not steady, some may even complain of blurred vision.’ (P3, Female, 59 years)

‘Sometimes the woman may complain of abdominal pains, she may also look very restless.’ (P6, Female, 74 years)

‘Often the woman, will look tired.’ (P7, Female, 44 years)

‘The woman may look weak, and she will even be shaking to indicate that she has no energy.’ (P9, Female, 66 years)

Some participants indicated that there were traditional medications used to manage blood loss. They reported use of different concoctions that are given orally:

‘To buy *Rooibos* tea so that the woman can regain the strength or recover the blood that has been lost during birthing.’ (P1, Female, 63 years)

‘We do have medication called *Selentjane* [*is red in colour*], and it is often given to the woman who is suspected to have lost too much blood.’ (P3, Female, 59 years)

‘We call *Moroto oa Lipela* [*urine of a Rock-Rabit*], an important medication because it helps even to heal wounds. We also give what is called *Letsoku* [*Red soil*].’ (P6, Female, 74 years)

‘The *Qobo and Mosisili* that I talked about earlier, this concoction is very strong to prevent blood loss, and often the woman recovers very quickly.’ (P9, Female, 66 years)

### Theme 3: Support during the management of the placenta by Basotho traditional birth attendants

#### Sub-theme 3.1 Support available during the management of the placenta

Participants indicated that whenever support was needed during the management of the placenta, the affected TBA would call a colleague to assist. In situations where the condition was very serious, they often asked for help from traditional healers; who normally succeeded in resolving such problems:

‘I normally call another traditional birth attendant and if she is not around, I normally call a traditional healer if she or he is around.’ (P4, Female, 62 years)

‘We normally call a traditional healer to assist, and they normally offer something for the woman to drink, and often the problem is solved.’ (P5, Female, 70 years)

Participants reported that it was important to have an adult woman or a family member present during the delivery and assist with preparations of things such as water and herbal medications needed to assist the birthing woman:

‘I always find it save when one member of the family is around so that she can run around when there is a need.’ (P3, Female, 59 years)

‘I normally call other adult women to come and help by making sure that there is warm water and preparing the medications that we may need.’ (P5, Female, 70 years)

#### Subtheme 3.2: Support needed to ensure safe management of the placenta

The participants indicated that they would perform their work safer for themselves, the mother, and the baby, if they could be supported with supplies such as gloves, razor blades, cotton wool, and plastic aprons. Others reported that they needed second-hand clothing since some women they assisted were very poor and did not prepare for the newborn before going into labour:

‘We would like to be assisted with things like gloves, or plastic linen protectors.’ (P1, Female, 63 years)

‘To be provided with kits that have cotton wool, linen savers, aprons, we do not have anything to protect ourselves with things like gloves or razors.’ (P5, Female, 70 years)

‘Things like aprons to protect ourselves, we would also want to be supported with gloves. we would like to be supported with equipment like soaps, and some second-hand clothing, as most women whom we assist are so poor that sometimes one does not have anything to cloth the baby, to an extent that we often offer our own clothing to cover the baby.’ (P9, Female, 66 years)

Some indicated the need for enforcing good relationships with health facilities and, most importantly, training to ensure safe practices while managing the third stage of labour:

‘Trainings are conducted for us time and again to be updated because these days …’ (P1, Female, 63 years)

‘Have a working relationship with people from Health.’ (P2, Female, 62 years)

‘Since these days the Ministry of Health is not supporting our practice, maybe we could be given frequent pieces of training on how we should safely manage our work.’ (P4, Female, 62 years)

‘If people from health could be interested to give us some training so that we know what is right.’ (P5, Female, 70 years)

‘We would like to have training because in our own culture, we have medications that we use, but we need training because it is in those training where we will be taught things like hygiene and other important things.’ (P7, Female, 44 years)

‘Periodic training where we can come together and learn the same things at the same time to make our work safer.’ (P8, Female, 68 years)

### Theme 4: Management during emergencies

The Basotho TBAs had measures they undertake to manage emergencies. This theme had one sub-theme.

#### Sub-theme 4.1: Measures taken by the Basotho traditional birth attendants during an emergency

Participants indicated that whenever they experienced serious third stage of labour emergencies they could not handle, they referred the woman to the nearest health facility. Referrals to health facilities were done with the help of relatives:

‘I always ask the relatives to take the woman to the nearest health facility. We never wait for too long or for sunset, we actually transfer the woman immediately.’ (P3, Female, 59 years)

‘I usually advise the family to take the woman to the nearest health facility.’ (P6, Female, 74 years)

‘Find transport to send a woman to the hospital though really all clinics are very far.’ (P5, Female, 70 years)

‘We normally refer the woman to the nearest clinic.’ (P7, Female, 44 years)

‘The woman is referred to the health facility, especially when I see that there is a problem which I cannot handle.’ (P8, Female, 68 years)

## Discussion

Basotho TBAs have their indigenous strategies for managing delivery of the placenta. These strategies involve blowing an empty bottle to assist with placenta expulsion, compression of the abdomen, application of controlled movements accompanied by whistle-blowing, and use of grass called *Loli*, which is rolled around the umbilical cord. In contrast to these practices, contemporary methods include active management of the third stage of labour, which involves the administration of uterotonic medication, controlled cord traction (CCT) as well as massaging of the birthing woman’s uterus (WHO 2017:C102). The WHO (2017:C103) advises that cord traction (pull) should not be applied without applying counter traction (push) above the pubic bone. Additionally, CCT is further discouraged without the presence of a skilled birth attendant. Similarly, in a study carried out in Ghana (Aziato & Omenyo 2018), TBAs were reported to be using indigenous practices to assist in the smooth delivery of the placenta. They provided a quote, ‘… we just cut plantain leaves and place it on the floor. When you place the woman on it, within 5 min the placenta will come out’ (Aziato & Omenyo 2018). Furthermore, in a study conducted in Kenya by Kaingu, Oduma and Kanui (2011), 32% of TBAs removed the placenta manually; about 6% massaged the lower abdomen, while 3% chose to refer patients to the hospital for further management.

Participants in this study indicated that traditionally, the placenta is expected to be delivered immediately after the baby’s birth. Although they considered 10–15 min as the normal waiting time for the placenta to be delivered, they could wait up to 1 h. This shows that the participants were aware of the normal time allowed for delivery of the placenta. Perlman and Carusi (2019:527) affirm that a placenta is considered retained when it does not spontaneously detach between 18 min and an hour after delivery of the infant. A birthing mother has a high chance of experiencing postpartum haemorrhage (PPH) and surgical morbidity as a result of a retained placenta, post-delivery of the baby (Perlman & Carusi 2019:533).

In instances where measures to assist expulsion of the placenta have failed, Basotho TBAs resort to using indigenous medicines. Indigenous medicines such as *Qobo (wild rhubarb)* and *Khomo ea Balisa (Bulbine narcissifolia)* are preferred medicines that are usually given to birthing mothers. It is believed these medicines help expel the placenta as well as the blood clots. Similarly, Kaingu et al. (2011:497) reported that more than half (56%) of TBAs used herbal medicine to assist delivery of the placenta. According to Shewamene, Dune and Smith (2017:382), medicinal plants are commonly used by African women for reproductive health indications as these medicines are easily accessible, unlike modern healthcare services where medicines are often out of stock. Falah and Hadiwibowo (2017:53), in their study conducted in Indonesia, also affirmed the wide usage of medicinal plants, such as *Parameria* and Macaranga winkleri, which are used to firm vaginal muscles and strengthen the uterus immediately after giving birth.

In this study, the Basotho TBAs indicated that they delivered women on the floor covered with blankets or sheepskin. In contrast, in a study carried out in India, although women were also reported to deliver on the floor, they were usually covered with dried long grass and old sacks, while others reported that the place of delivery was covered with cow dung (Saxena, Jelly & Sharma 2020:158). The TBAs were cognisant of blood loss during the third stage of labour; therefore, blood was collected from all the materials that the birthing woman is made to sleep on to observe the amount of blood loss. Prevention of blood loss is an important issue for Basotho TBAs. In the current study, participants reported that blood overflowing the sheepskin or blanket used by the woman to lie on during the birthing process indicated that the woman has lost too much blood. In contrast to a study conducted by Ononge, Okello and Mirembe (2016:211), where the TBAs measured the amount of blood loss with cups to estimate if the woman had PPH, and if they suspected that the bleeding was more than normal, an acceptable amount, they referred the mother to the healthcare facility.

Traditionally, a variety of indigenous medicinal plants or concoctions are prepared before a woman gives birth as an emergency preparedness measure; in case the woman shows signs of too much blood loss after delivery of the placenta. In the case that a woman showed signs of bleeding, the medicinal plants were given to the woman to drink to stop the bleeding. In this study, participants offered birthing mothers either *rooibos, Selentjane* (red in colour), *Qobo* (wild rhubarb), *Letsoku* (red soil) or Moroto or Lipela (urine of a Rock-Rabit) to stop excessive bleeding. Antidiabetic activity has been reported from using *Qobo* (Balogun, Tshabalala & Ashafa 2016). It is acceptable in modern practice to use oxytocin or misoprostol to prevent PPH; however, since oxytocin must be administered intramuscularly, the practitioner needs to be trained in how to inject it (WHO 2012:5).

Participants reported positioning during birthing as important to prevent blood loss. The woman usually assumed a squatting position and rested on a traditionally made ring (*Khare*) between her heels and the anus to avoid tearing and bleeding. This is similar to the findings in a study conducted by Saxena et al. (2020:158) in India, where most women (92%) birthed in a sitting position; however, this position may not be favourable for delivery as chances of rupture of the pelvic floor muscles are increased. The seating position is associated with an increased blood loss of 500 mLs–to 1000 mLs, whereas squatting among nulliparous Turkish women was associated with a shortened second stage of labour (Moraloglu et al. 2017:245; Thies-Lagergren et al. 2011:22).

The participants in the study indicated that they transferred women to a healthcare facility in cases of complications beyond their control. Similarly, in studies conducted in Ethiopia and Ghana, there was no formal relationship between TBA and healthcare facilities; thus, TBAs only referred women to health facilities if they faced problems such as excess bleeding or retained placenta during the process of birth (Adatara et al. 2018:354; Kassie et al. 2022:425).

Participants reported that the Lesotho Ministry of Health no longer support their traditional practices. However, because of the inaccessibility of health facilities, TBAs still assist women during birthing. Despite TBAs being discouraged from attending to deliveries at home, some TBAs continue to help women during birth as women require their assistance (Wilunda et al. 2017:e0185725).

Traditional birth attendants reported that because of current illnesses such as HIV and/or AIDS that are prevalent these days, the use of protective clothing is very important, and equipment such as gloves are needed to avoid handling clients with bare hands. They are also in need of razor blades or scissors to separate the baby from the placenta. Participants further indicated the importance of a nearby health facility for easy referral of women whenever a need arose.

Participants indicated their willingness to work in collaboration with staff from health facilities and the need for refresher training on issues such as hygiene and other safety measures that need to be implemented in the management of women during delivery of the placenta. In some countries, training of TBAs has been shown to increase the number of hospital referrals when obstetric complications occur in comparison to non-trained birth attendants, thus reducing maternal morbidity and mortality rates (Amutah-Onukagha et al. 2017:131). The training of TBAs improved not only skills but also knowledge of identification of danger signs accurately (MacDonald 2017:020302; Shimpuku et al. 2021:137).

## Limitations of the study

This study was conducted in only two rural villages of Quthing District that were purposively selected as they have many TBAs and were accessible by road despite the moderate snowfall. Subsequently, this study cannot be considered representative of the entire population of Lesotho practicing TBAs. However, its findings provide an acceptable picture of how Basotho TBAs manage the third stage of labour.

## Conclusion

Traditional birth attendants continue to manage the third stage of labour despite the challenges of being unwanted and a lack of support for their work. The participants displayed knowledge of danger signs during the third stage of labour. Some of their practices such as requesting a woman to blow a bottle while pushing the placenta are relevant because pressure exerted on the muscles helps to propel the placenta to be delivered. They remain accessible to the women in their community compared to the distances women must travel to access modern health care. The medicinal plants used by the TBAs during pregnancy, delivery and third stage are easily accessible compared to contemporary medicines; however, the medicinal plants they use have not been studied for toxicity and effectiveness.
